# Association of fertility diet score with endometriosis: a case–control study

**DOI:** 10.3389/fnut.2023.1222018

**Published:** 2023-09-01

**Authors:** Sakine Ghasemisedaghat, Ghazaleh Eslamian, Seyyedeh Neda Kazemi, Bahram Rashidkhani, Robabeh Taheripanah

**Affiliations:** ^1^Department of Cellular and Molecular Nutrition, Faculty of Nutrition and Food Technology, National Nutrition and Food Technology Research Institute, Shahid Beheshti University of Medical Sciences, Tehran, Iran; ^2^Department of Obstetrics and Gynecology, School of Medicine, Preventative Gynecology Research Center, Imam Hossein Hospital, Shahid Beheshti University of Medical Sciences, Tehran, Iran; ^3^Department of Community Nutrition, Faculty of Nutrition and Food Technology, National Nutrition and Food Technology Research Institute, Shahid Beheshti University of Medical Sciences, Tehran, Iran; ^4^Department of Obstetrics and Gynecology, School of Medicine, Men's Health and Reproductive Health Research Center, Imam Hossein Hospital, Shahid Beheshti University of Medical Sciences, Tehran, Iran

**Keywords:** fertility diet score, dietary assessment, endometriosis, women, case-control

## Abstract

**Background and aims:**

Different factors, such as environmental, epigenetic, genetic and immunological, have been identified as potential risks for developing endometriosis. However, the correlation between dietary patterns and endometriosis is currently unknown. The aim of this study was to explore the potential link between fertility diet score and the odds of endometriosis.

**Methods:**

This study was a hospital-based case–control study that took place in a gynecology clinic in Tehran, Iran, between February 2021 and January 2022. A total of 107 newly diagnosed endometriosis cases and 210 controls were included. The participants' habitual diets were evaluated using a food frequency questionnaire, and their fertility diet score was estimated using a point system based on Chavarro et al.'s criteria. The logistic regression was utilized to calculate the odds ratios (OR) with 95% confidence intervals (CIs).

**Results:**

The study found that women who adherence to fertility diet have a lower odds of endometriosis. This was observed in both the base model and the adjusted model, with a significant decrease in odds of endometriosis by 66% (OR = 0.44, 95%CI = 0.27–0.71, *p* = 0.001) and 54% (aOR = 0.46, 95%CI = 0.23–0.90, *p* = 0.022), respectively. Additionally, consuming vegetable proteins and multivitamins were also associated with lower odds of endometriosis. On the other hand, consuming animal proteins, heme iron, and having a high glycemic load were associated with significantly higher odds of endometriosis.

**Conclusion:**

Our research supports the hypothesis that following a fertility diet may decrease the odds of endometriosis in Iranian women. However, these findings should be verified through extensive, prospective studies.

## 1. Introduction

Endometriosis is an estrogen-dependent chronic inflammatory gynecological disease (1) that affects about 10% of women in their childbearing years (2). This condition is caused by the presence of endometrial tissue (glands and stroma) beyond the uterine cavity, which can distort the pelvic anatomy of women (1, 3). Common consequences of endometriosis include chronic pelvic pain, dysmenorrhea, dyspareunia, dysuria, dyschezia, subfertility, and infertility (4, 5). These associated symptoms can significantly reduce the quality of life for affected women (6) and can affect health, education, relationships, employment and performance (6–8).

Despite extensive research on endometriosis, its exact cause remains unclear (9). The origins of endometriosis vary greatly among different women (9, 10). Identified risk factors for endometriosis includes environmental, epigenetic, genetic and immunological (10). Within the category of environmental factors, dietary risk factors, such as high intakes of fats, trans-unsaturated fatty acids, coffee, red meat, and alcohol, have been reported to increase the risk of endometriosis (11, 12). On the other hand, adhering to a diet rich in fruits, vegetables, fish oils, and calcium-, vitamin C-, and vitamin D-rich dairy products may inversely be associated with endometriosis (11, 12). Therefore, emerging evidence suggests that dietary intake is a potentially modifiable risk factor for endometriosis (13). Previous studies have demonstrated that oestrogen activity, inflammation and prostaglandin metabolism are related to endometriosis, although the mechanisms are vague (13).

At present, there is a lack of information about the relationship between dietary indices and endometriosis, and no research has been conducted to determine the correlation between fertility diet score and endometriosis. The fertility diet score evaluates the combined effects of various nutrients, foods, and supplements (14). This dietary pattern, as determined by the fertility diet score, is based on research conducted by Chavarro et al. using data from the US Nurses' Health Study. Their findings suggest a connection between the risk of ovulatory dysfunction and specific dietary factors. A high fertility diet score is characterized by a lower consumption of trans fat and a higher intake of monounsaturated fat. It also involves a reduced intake of animal protein and an increased intake of vegetable protein. The diet emphasizes a higher consumption of high-fiber, low-glycemic carbohydrates, a preference for high-fat dairy products, a higher intake of nonheme iron, and a greater frequency of multivitamin use. Following this fertility dietary pattern may positively impact reproductive health (14).

Therefore, the aim of the present study was to investigate the association between fertility diet score and the odds of endometriosis among Iranian women.

## 2. Methods

### 2.1. Ethical considerations

The study, in accordance with the Declaration of Helsinki (15), was authorized by the Medical Ethical Committee of Shahid Beheshti University of Medical Sciences' National Nutrition and Food Technology Research Institute (IR.SBMU.NNFTRI.REC.1399.062) in Iran. Written consent was obtained from all participants, and confidentiality of data was ensured.

### 2.2. Study design and population

This is a hospital-based case–control study that involved face-to-face interviews with all participants. Between February and September 2021, we interviewed 115 consecutive, newly diagnosed endometriosis patients (within 6 months) and 230 controls. Women cases were eligible if they were aged at 18–49, non-pregnant, non-lactating and non-menopausal and were not afflicted with diet related chronic diseases such as diabetes, cardiovascular disease, renal failure, cancer, etc. A case was considered as any patient diagnosed with endometriosis, based on surgery, macroscopically or with histological examination. The control group had the same inclusion criteria as the case group, except for the presence of endometriosis. Suitable control participants were women who did not have endometriosis or pelvic pain syndrome. All cases and controls were confirmed by a gynecologist who did not know the objectives of the study.

### 2.3. Dietary intake assessment

Dietary intake was assessed by the previously validated food frequency questionnaire (FFQ) (16). FFQs serve the purpose of assessing long-term nutrient intake patterns and can identify aspects of an individual's diet that may not have been captured in a short-term recall. Our FFQ contained 168 foods and beverages (with standard serving sizes) regularly consumed by Iranians. Diets of participants were based on intakes during the year prior to endometriosis diagnosis (cases) or interview (controls). The participants in the interview were informed about the average size of each food item. In order to assist participants to evaluate type of food items and the portion size, a validated food album (17) and images of household measurements were used. After that, they provided information on how often they consume each food item in a day, week, or month. These consumption values were converted to grams using a reference scale for measuring food at home. The average daily intake of energy and macronutrients was calculated using data from the USDA food composition table (18) or the Iranian food composition table (19). To prevent information bias, a single interviewer conducted all interviews and questionnaires without prior knowledge of the participants' outcomes.

The fertility diet score that ranged from 8 to 40 point was calculated by allocating points to each dietary factors including high fat dairy, vegetable protein, increased monounsaturated/ trans-fat (MUFA/TFA) ratio, multivitamins, iron, low fat dairy, animal protein and glycemic load based on Chavarro et al. (14). Higher fertility diet score reflected higher adherence to the fertility diet.

### 2.4. Non-dietary exposure assessment

The researchers gathered data on various factors, including age, number of pregnancies, parity, monthly income, smoking habits, endometriosis history in the family, education level, occupational status, and use of multivitamins. They also recorded the height, weight, and waist circumference of the participants following established protocols and calculated their body mass index (BMI) using weight and height measurements. To measure physical activity, they used a valid and reliable questionnaire (20). It is a tool created by Aadahl et al. to assess overall physical activity in a day, encompassing sleep, work, and free time on a typical weekday (20).

### 2.5. Statistical analysis

Statistical analysis was conducted using SPSS (Statistical Package for the Social Sciences program; version 20; Chicago, IL, United States). All analyses were two-tailed, with *p*-values <0.05 considered statistically significant. The continuous variables were tested for normal distribution using Q-Q plots and the Shapiro–Wilk test before conducting further tests. Frequency and percentages were used to describe the categorical demographic characteristics, while the median (interquartile range, IQR) was used for quantitative characteristics. The Chi-Square test was employed to compare qualitative data between endometriosis patients and controls. To compare quantitative features between the two groups, the Mann–Whitney test was utilized. The fertility diet score and its components were categorized based on the median for each item in the control group. Binary logistic regression was employed to assess the relationship between the fertility diet score and endometriosis and calculate adjusted odds ratios (OR) with a 95% confidence interval (CI). All food components were adjusted for energy using the residual method. Additionally, age (years), occupation, BMI (Kg/m2), smoking (yes/no), fat (g/day) and familial history of endometriosis (yes/no) were controlled for adjusted ORs.

## 3. Results

General characteristics of the case and control participants are described in Table 1. There was not a significant difference in age of control (32 [29–36]) and case (33 [25–39]) group participants (*p* = 0.819). Cases had significantly higher family history of endometriosis (*p* < 0.001). Cases and controls were not significantly different according to age at menarche (*p* = 0.306), marital status (*p* = 0.436), physical activity (*p* = 0.844), BMI (*p* = 0.072), WC (*p* = 0.902), number of pregnancy (*p* = 0.684), education (*p* = 0.870) and occupation status (*p* = 0.057). A significant distinction was observed between the case and control groups based on their BMI classification (*p* = 0.005). The percentage of individuals who were underweight, overweight, and obese was higher in the case group compared to the control group. Conversely, the percentage of individuals with a normal BMI was higher in the control group than in the case group.

**Table 1:** General characteristics of participants with endometriosis and controls.

Characteristic^*^	Endometriosis women *n* = 107	Control women *n* = 210	*P* Value^†^
Age, year	33 (25–39)	32 (29–36)	0.819
Age at menarche, year	13 (11–15)	13 (12–14)	0.306
Marital status, No (%)			0.436
Single	43 (40.2)	75 (35.7)	
Married	64 (59.8)	135 (64.3)	
Familial history of Endometriosis, No (%)			<0.001
Yes	33 (30.8)	6 (2.9)	
No	74 (69.2)	204 (97.1)	
Physical activity (MET/h/d)	27 (21–36)	26 (21–48)	0.844
Body Mass Index (kg/m^2^)	26.9 (22.8–29.9)	24.9 (21.8–28.9)	0.072
Body Mass Index Classification, No (%)			0.005
Underweight	14 (13.1)	12 (5.7)	
Normal weight	29 (27.1)	96 (45.7)	
Overweight	39 (36.4)	59 (28.1)	
Obese	25 (23.4)	43 (20.5)	
Waist circumference (cm)	82 (76–93)	85 (75–93)	0.902
Education, No (%)			0.961
Primary/secondary school	36 (33.6)	70 (33.3)	
Bachelor’s degree	53 (49.5)	102 (48.6)	
Master’s/Doctoral degree	18 (16.8)	38 (18.1)	
Cigarette smoker, No (%)			0.002
Past smokers	28 (26.2)	32 (15.2)	
Never smokers	55 (51.4)	116 (55.2)	
Smoker (1–2 cigarette/d)	3 (2.8)	30 (14.3)	
Ex-smokers (≥3 cigarette/d)	21 (19.6)	32 (15.2)	
Occupation, No (%)			0.057
Employed	46 (43)	114 (54.2)	
Housewife	61 (57)	96 (45.8)	
Number of pregnancy, No (%)			0.684
0	59 (55.1)	105 (50)	
1–2	36 (33.7)	78 (37.1)	
≥3	12 (11.2)	27 (12.9)	

* Values are median (IQR) unless otherwise noted.

† Using Mann Whitney test or *χ*^2^ test, as appropriate.

Characteristics of dietary intakes of both groups are shown in Table 2. The Median [IQR] fertility diet score in the case group [20 (18–23)] was significantly lower than that in the control group [21 (19–24)] (*p* = 0.007).

**Table 2:** Dietary intakes among endometriosis cases and controls.

Dietary factors^*^	Endometriosis women *n* = 107	Control women *n* = 210	*P* Value^†^
Energy intake (kcal/d)	2145.6 (1753.2–3091.4)	2036.7 (1523.2–2575.1)	0.2
Carbohydrate (g/d)	269.9 (207.2–374.4)	307.6 (221.2–392.3)	0.039
Protein (g/d)	76.3 (58.9–101.4)	68.6 (52.5–89.4)	0.004
Fat (g/d)	94.4 (66.3–141.9)	61.5 (46.6–84.6)	<0.001
Dietary fiber (g/d)	14 (11.1–19.8)	25.8 (16.3–35)	<0.001
Saturate fatty Acid (g/d)	33.9 (20.3–48.6)	18.1 (13.23–25.04)	<0.001
Unsaturated fatty Acid (g/d)	20.3 (14.4–27)	19.3 (13.8–25.8)	0.350
Fertility diet score	20 (18–23)	21 (19–24)	0.007

* Values are median (Q1–Q3).

† Based on Mann Whitney test.

Table 3 displays the association between the fertility diet score and its individual components with odds of endometriosis. According to the base model, women who scored in the upper median of fertility diet were observed to have significantly lower odds of endometriosis, specifically by 66% (OR = 0.44, 95%CI = 0.27–0.71, *p* = 0.001). This significant association remained in the adjusted model (aOR = 0.46, 95%CI = 0.23–0.90, *p* = 0.022). Regarding fertility diet components, the odds of endometriosis were 85 and 82% lower in the upper median of vegetable proteins in both the base and adjusted models (OR = 0.15, 95%CI = 0.09–0.29, *p* < 0.001, and aOR = 0.18, 95%CI = 0.11–0.32 *p* < 0.001, respectively). High consumption of multivitamins was associated with 73 and 69% lower odds of endometriosis in base and adjusted models (OR = 0.27, 95%CI = 0.16–0.43, *p* < 0.001, and aOR = 0.31, 95%CI = 0.13–0.52, *p* < 0.001, respectively). In the base model, a significant direct association was observed between odds of endometriosis and high consumption of animal proteins (OR = 5.97, 95%CI = 2.34–8.37, *p* < 0.001), heme iron (OR = 5.05, 95%CI 1.22–7.21, *p* < 0.001) and glycemic load (OR = 2.49, 95%CI = 1.31–3.21, *p* = 0.004). In the adjusted model, endometriosis odds was also higher in the upper median of consumption of animal proteins (aOR = 5.11, 95%CI = 2.61–7.91, *p* < 0.001), heme iron (aOR = 5.48, 95%CI = 1.72–7.81, *p* < 0.001) and glycemic load (aOR = 2.44, 95%CI = 1.22–3.90, *p* = 0.024). Although participants in the upper median of MUFA/TFA ratio had lower odds of endometriosis, this association was not statistically significant. In both the base and adjusted models, the association between high-fat dairy and low-fat dairy with endometriosis was not statistically significant.

**Table 3:** Association between fertility diet score and endometriosis odds: adjusted odds ratio (OR) estimates and 95% confidence intervals (CIs)^*^.

Fertility diet score and its components	Median of score or dietary components		*P* Value†
	< median	≥ median	
Fertility diet score			
No. Cases/No. Controls	113:71	97:36	
Base model^†^	1.00 (Ref.)	0.44 (0.27–0.71)	0.001
Full model^‡^	1.00 (Ref.)	0.46 (0.23–0.90)	0.022
Ratio of monounsaturated to trans fat			
No. Cases/No. Controls	110:62	100:45	
Base model^†^	1.00 (Ref.)	0.80 (0.50–1.28)	0.347
Full model^‡^	1.00 (Ref.)	0.85 (0.45–1.64)	0.634
Animal protein			
No. Cases/No. Controls	139:7	71:100	
Base model^†^	1.00 (Ref.)	5.97 (2.34–8.37)	<0.001
Full model^‡^	1.00 (Ref.)	5.11 (2.61–7.91)	<0.001
Vegetable protein			
No. Cases/No. Controls	103:87	107:20	
Base model^†^	1.00 (Ref.)	0.15 (0.09–0.29)	<0.001
Full model^‡^	1.00 (Ref.)	0.18 (0.11–0.32)	<0.001
Glycemic load			
No. Cases/No. Controls	91:42	119:65	
Base model^†^	1.00 (Ref.)	2.49 (1.31–3.21)	0.004
Full model^‡^	1.00 (Ref.)	2.44 (1.22–3.90)	0.024
Multivitamins			
No. Cases/No. Controls	76:73	134:34	
Base model^†^	1.00 (Ref.)	0.27 (0.16–0.43)	<0.001
Full model^‡^	1.00 (Ref.)	0.31 (0.13–0.52)	<0.001
Heme Iron			
No. Cases/No. Controls	89:10	121:97	
Base model^†^	1.00 (Ref.)	5.05 (1.22–7.21)	<0.001
Full model^‡^	1.00 (Ref.)	5.48 (1.72–7.81)	<0.001
High-fat dairy			
No. Cases/No. Controls	69:30	141:77	
Base model^†^	1.00 (Ref.)	1.26 (0.75–2.09)	0.382
Full model^‡^	1.00 (Ref.)	0.84 (0.11–4.70)	0.623
Low-fat dairy			
No. Cases/No. Controls	100:52	110:55	
Base model^†^	1.00 (Ref.)	0.96 (0.60–1.53)	0.869
Full model^‡^	1.00 (Ref.)	0.94 (0.77–3.15)	0.857

^*^Logistic regression model.

^†^Adjusted for age.

^‡^Adjusted for age, occupation, smoking, familial history of endometriosis, fat, fiber, BMI.

## 4. Discussion

### 4.1. Main finding

To the best of our knowledge, this study is the first to investigate the association between fertility diet score and endometriosis in a developing country. The findings revealed an inverse relationship between endometriosis and fertility diet score, highlighting the importance of high intake of vegetable proteins and multivitamins. Conversely, we observed a positive association between endometriosis and consumption of animal protein, heme iron, and glycemic load.

### 4.2. Interpretation

According to our study, a diet with a higher fertility score was associated with lower odds of endometriosis. However, since there have been no previous studies on the correlation between this score and endometriosis, we compared our findings to studies regarding other outcomes. Two previous studies have shown a negative correlation between the fertility diet score and both polycystic ovary syndrome (21) and ovulatory disorder infertility (14). However, a study by Gaskins et al. found that adhering to a fertility diet did not increase the chances of a successful live birth through assisted reproductive technologies (22).

According to our research, there is a correlation between consuming high amounts of vegetable proteins and a lower odds of endometriosis. However, previous studies have not found any significant difference in the consumption of vegetable proteins between individuals with endometriosis and those without (23, 24). One study revealed that consuming more vegetables in general was correlated with a lower risk of developing endometriosis (25). However, another study reported that there was no association between vegetable intake and the risk of endometriosis (24).

Our research suggests that taking multivitamins may be protective against endometriosis. This may be due to the fact that lower levels of vitamin D, zinc, and vitamin E are associated with a higher risk for endometriosis (26). A review of *in vitro*, animal, and human studies suggests that dietary supplements could be considered a complementary treatment option for endometriosis (26).

Based on our research, it was discovered that the odds of developing endometriosis is higher among those who consume animal protein and heme iron. This finding contradicts the report by Samaneh et al. (25), which suggests that women with lower animal protein intake are more likely to have endometriosis. Although there have been limited studies investigating the relationship between animal protein and endometriosis, we examined the findings of our study alongside those examining the correlation between dietary sources of animal proteins and endometriosis. Our study aligns with Parazzini et al.'s research (12), which revealed an increased risk of endometriosis associated with the consumption of red meat. However, Heilier et al. (27) and Trabert et al. (24) did not find any statistically significant correlation between the consumption of red meat and the occurrence of endometriosis. The higher occurrence of saturated fatty acids, heme iron, and animal proteins in animal-based foods may explain this correlation. Saturated fats, found primarily in animal products, may lead to elevated plasma levels of oestradiol or steroid hormones, leading to an increased risk of oestrogen-related diseases.

Our study found a direct association between glycemic load and endometriosis, which contrasts with the findings of Schwartz et al.'s study that showed no such correlation among premenopausal women (28). A high glycemic load diet typically consists of low levels of complex carbohydrates and fiber. In our study, we noticed that the group with endometriosis consumed significantly less dietary fiber compared to the control group. This could be explained by the potential risk of endometrial proliferation and hence endometriosis from consuming simple carbohydrates with a high glycemic index (29). Contrasting our findings, Savaris et al. (30) found that increasing fiber intake was associated with a higher risk of endometriosis, but this study had a small sample size and cannot be generalized. Additionally, Trabert et al. (24) and Britton et al. (31) were unable to establish a significant relationship between a high-fiber diet and endometriosis.

Although participants with a high intake of MUFA/TFA ratio had reduced odds of developing endometriosis, the association was not statistically significant in our study. Similar to our findings, Trabert et al. (24) also did not find any significant impact of increased consumption of trans fats on the risk of developing endometriosis. Studies conducted by Parazzini et al. (32) and Heilier et al. (27) found no link between the consumption of trans fats through margarine and the occurrence of endometriosis. In contrast, Missmer et al. (33) demonstrated that women who consumed the highest amounts of trans fats had a greater odds of endometriosis than women who consumed the lowest amounts of trans fats. In line with our study, no previous studies have established any definite links regarding the potential impact of MUFA on the risk of endometriosis (24, 30, 33).

The current research determined that there is no notable correlation between the intake of high-fat dairy or low-fat dairy and endometriosis. This result is in line with the findings of Parazzini et al. (32) and Heilier et al. (27), who could not establish an association between milk or cheese and the occurrence of endometriosis. In contrast, Harris et al. demonstrated that the consumption of low-fat milk and other dairy products was linked to a reduced risk of endometriosis (34). Specifically, their findings indicated that the consumption of more than three servings of dairy products per day was associated with an 18% lower risk than the consumption of only two servings (34). The disparities between our study and Harris et al.'s may be due to the lower *per capita* consumption of dairy products in Iran, where the average daily intake in our study was less than 1.5 servings.

We compared our results in relation to similar studies conducted in different countries. There are reasons that can account for the differences we observed. These variations in eating habits among countries can be attributed to cultural factors, historical practices, and socio-economic conditions. Although globalization has brought about some level of standardization in dietary patterns in certain regions, traditional diets continue to thrive in many countries. This is because these dietary traditions are ingrained within the culture and are passed down through generations, fostering a strong sense of cultural identity and influencing personal food choices.

### 4.3. Strengths and limitations

The outcomes of our study may have been affected by certain limitations. It is conceivable that participants who were already aware of their endometriosis condition before attending the study clinic may have remembered their dietary intake differently from the control group. Although efforts were made by researchers to prevent bias, some types of bias such as selection bias, measurement bias, and recall bias could still lead to erroneous conclusions when using a case–control method. Another limitation was the insufficient data available on the clinical stage of the disease for all participants, highlighting the potential impact it may have on the understanding of the relationship between diet and the disease process in women with endometriosis. Additionally, another limitation of our study was the omission of data related to infertility or the use of assisted reproductive technologies in our analysis.

However, there were also several strengths in our research. We achieved high rates of participation from both cases and controls, and we had access to several potential variables for adjusting our regression models. To ensure data quality, we excluded participants who provided incorrect or excessive energy intake information and patients who had not received a diagnosis within the past 6 months. Our selection of incident cases reduced recall bias and strengthened the interpretation of causality. We employed a validated FFQ to evaluate exposure, and a professional dietitian who remained unaware of participants' diagnostic status conducted the interviews.

## 5. Conclusion

This study revealed that a greater adherence to the fertility diet, indicated by a higher fertility diet score, showed a negative association with endometriosis among Iranian women. Our results suggest that interventions promoting the fertility diet could be an effective method for endometriosis prevention. Additional prospective studies are required to confirm these findings.

## Data availability statement

The raw data supporting the conclusions of this article will be made available by the authors, without undue reservation.

## Ethics statement

The studies involving humans were approved by the Ethical Committee of Shahid Beheshti University of Medical Sciences' National Nutrition and Food Technology Research Institute (IR.SBMU.NNFTRI.REC.1399.062). The studies were conducted in accordance with the local legislation and institutional requirements. The participants provided their written informed consent to participate in this study.

## Author contributions

GE: conceptualization and supervision. SG and GE: data curation, writing—review and editing, software, formal analysis, and writing—original draft preparation. SK and RT: investigation. GE, BR, and SK: methodology. SK and BR: visualization. RT and BR: validation. All authors contributed to the article and approved the submitted version.

## Funding

The study was supported by grant NO 99/25850 from the National Nutrition and Food Technology Research Institute, Shahid Beheshti University of Medical Sciences, Tehran, Iran.

## Conflict of interest

The authors declare that the research was conducted in the absence of any commercial or financial relationships that could be construed as a potential conflict of interest.

## Publisher’s note

All claims expressed in this article are solely those of the authors and do not necessarily represent those of their affiliated organizations, or those of the publisher, the editors and the reviewers. Any product that may be evaluated in this article, or claim that may be made by its manufacturer, is not guaranteed or endorsed by the publisher.
